# Hyaluronan-CD44 interaction promotes c-Jun signaling and miRNA21 expression leading to Bcl-2 expression and chemoresistance in breast cancer cells

**DOI:** 10.1186/1476-4598-13-52

**Published:** 2014-03-08

**Authors:** Liqun Chen, Lilly Y W Bourguignon

**Affiliations:** 1 San Francisco Veterans Affairs Medical Center and Department of Medicine, University of California at San Francisco & Endocrine Unit (111N2), 4150 Clement Street, San Francisco, CA 94121, USA

## Abstract

MicroRNA-21 (miR-21) is associated with the development of solid tumors progression including breast cancer. In this study we investigated matrix hyaluronan (HA)-CD44 (a primary HA receptor) interaction with c-Jun N-Terminal Kinase (JNK)/c-Jun signaling in MDA-MB-468 breast cancer cells [a triple-negative (estrogen receptor-negative/progesterone receptor-negative/HER2-negative) breast cancer cell line]. Our results indicated that HA binding to CD44 promotes c-Jun nuclear translocation and transcriptional activation. Further analyses revealed that miR-21 is regulated by an upstream promoter containing AP1 binding site(s), and chromatin immunoprecipitation (CHIP) assays demonstrated that stimulation of miR-21 expression by HA/CD44 interaction is c-Jun-dependent in these breast cancer cells. This process results in an increase of the anti-apoptosis protein Bcl-2 and upregulation of inhibitors of the apoptosis family of proteins (IAPs) as well as chemoresistance in MDA-MB-468 cells. Treatment with c-Jun specific small interfering RNAs effectively blocks HA-mediated c-Jun signaling and abrogates miR-21 production as well as causes downregulation of survival proteins (Bcl-2 and IAPs) and enhancement of chemosensitivity. In addition, our results demonstrated that anti-miR-21 inhibitor not only downregulates Bcl-2/IAP expression but also increases chemosensitivity in HA-treated breast cancer cells. Together, these findings suggest that the HA/CD44-induced c-Jun signaling plays a pivotal role in miR-21 production leading to survival protein (Bcl-2/IAP) upregulation and chemoresistance in triple negative breast cancer cells such as MDA-MB-468 cell line. This novel HA/CD44-mediated c-Jun signaling pathway and miR-21 production provide a new drug target for the future intervention strategies to treat breast cancer.

## Introduction

Matrix Hyaluronan (HA) is an anionic, nonsulfated glycosaminoglycan distributed widely throughout connective, epithelial, and neural tissues [1]. As a major component in the extracellular matrix of most mammalian tissues, HA contributes significantly to cell adhesion, proliferation and migration/invasion [2-4]. There is also a great deal of evidence linking high level of HA production in human carcinomas to aggressive phenotypes and metastasis, including the progression of breast cancer [2-7].

CD44 is a family of cell-surface glycoproteins that are expressed in a variety of tissues, including breast cancer tissues [2,3]. RHAMM whose cell surface form is now designated as CD168, was also found in breast cancer cells [8,9]. Both CD44 and RHAMM mediate hyaluronan signaling [10]. However, these two HA receptors likely regulate cellular signaling by different mechanisms because they are not homologous proteins, are compartmentalized differently in the cell, and differ in the way by which they bind to HA [10]. Since CD44 was identified as the first integral HA binding “receptor”, HA-mediated CD44 signaling has received a great deal of attention in cancer field. Both CD44 and HA are overexpressed/elevated at sites of tumor attachment [1,4]. HA binding to CD44 not only affects cell adhesion to extracellular matrix (ECM) components, but also stimulates a variety of tumor cell-specific functions leading to breast cancer progression [2,3,11-14]. However, the oncogenic mechanism(s) occurring during HA-activated and CD44-specific breast cancer progression remain(s) to be determined.

Jun N-terminal kinases (JNKs) belong to the mitogen-activated protein kinase family, and are responsive to stress stimuli, such as cytokines, ultraviolet irradiation, heat shock, and osmotic shock [15]. Activation of JNKs by targeting phosphorylation of downstream effector proteins (e.g., c-Jun, ATF2, ELK1, SMAD4, p53 and HSF1) leads to a number of important cellular functions including cell growth, differentiation, survival and apoptosis [15,16]. Among these JNK-regulated target proteins, c-Jun was initially identified as the c-Fos-binding protein. The association between c-Jun and c-Fos forms the AP-1 early response transcription factor complex which then binds to DNA sequences located in the promoter regions of genes stimulated by externally added agonists [17]. In human cancer, the level of *c-Jun* and c-*fos* mRNA and AP-1 expression has been shown to be elevated in drug-resistant tumor cells (such as etoposide resistant human leukemia K562 cells) as compared to the *c-Jun*/c-*fos* mRNA/AP-1 levels found in drug-sensitive parental lines [18]. Mitogenic stimulation of breast tumor cells (MCF-7 cell line) by insulin or insulin-like growth factors (IGFs) has been shown to promote c-Jun or c-*fos* upregulation and AP-1 activity [19]. Previous studies showed that persistent expression of c-Jun protein prevents stromal cells from entering apoptosis during the late secretory phase [20]. CD44 ligation blocks cell cycle progression of myeloid leukemia cells by downregulating c-Jun expression [20]. These observations suggest that c-Jun signaling is involved in regulating tumor cell growth, survival/anti-apoptosis and chemoresisitance.

MicroRNAs (miRNAs) are single-stranded RNAs of 21–25 nucleotides in length, which have been found to modulate gene expression at the posttranslational level [21]. MicroRNAs (miRNAs) are essential for normal development as modulators of gene expression. An estimated 30%-60% of the genome is regulated by miRNA-mediated silencing [22], however aberrant expression of miRNAs is associated with many diseases, including cancer. Recent studies indicate that that some microRNAs upregulate the expression of its target gene by binding to the 3′ UTR [23,24]. Overexpression of miR-21 influences cell proliferation, invasion, metastasis and chemoresistance in different cancer cells including breast cancer cells [25-27]. The identified targets of miR-21 in human cancer cells include a tumor suppressor protein [Program Cell Death 4 (PDCD4)] [28]. A previous study indicated that HA-CD44 interaction promotes miR-21 production, and PDCD4 reduction in both breast cancer cells (MCF-7 cell line) and head and neck cancer cells (HSC-3 cell line) [25,29]. This event contributes to upregulation of inhibitors of apoptosis proteins (IAPs) and the multidrug resistant protein (MDR1)/P-glycoprotein (P-gp) resulting in anti-apoptosis and chemotherapy resistance in breast tumor cells (MCF-7 cell line) [25]. Thus, miR-21 is currently considered to be an oncogene. A recent report indicates that miR-21 can also stimulate the expression of an anti-apoptosis protein, Bcl-2 by binding directly to the 3′UTR of Bcl-2 mRNA [24]. Upregulation of Bcl-2 expression by miR-21 is associated with anti-apoptosis, chemoresistance and proliferation in pancreatic cancer cells [24]. The question of whether Bcl-2 expression is associated with miR-21 production in HA-treated breast tumor cells has not been addressed.

In this study we investigated a new HA/CD44-mediated c-Jun signaling pathway that regulates miR-21 production and chemoresistance in MDA-MB-468 cell line (a triple negative breast cancer cell line). Our results indicated that HA/CD44 activates c-Jun signaling which, in turn, stimulates miR-21 expression and function. These events lead to the production of an anti-apoptosis protein, Bcl-2 and upregulation of survival proteins (IAPs) and Doxorubicin chemoresistance in MDA-MB-468 cells. cells. Inhibition of c-Jun signaling or silencing miR-21 expression/function not only results in Bcl-2 downregulation, but also causes a reduction of survival protein expression and enhances chemosensitivity to Doxorubicin. Thus, our findings strongly support the contention that HA/CD44-regulated c-Jun and miR-21 form a functional signaling axis that regulates tumor cell survival and Doxorubicin chemoresistance in triple negative breast cancer cells such as MDA-MB-468 cells.

## Results

### HA-CD44 interaction activates JNK and c-Jun signaling in breast tumor cells

**Figure 1:**
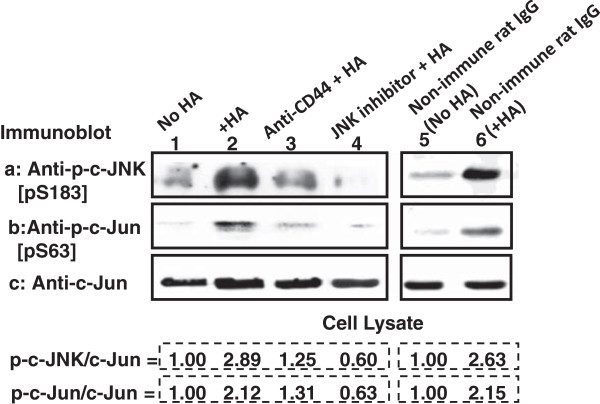
**Detection of HA/CD44-induced JNK and c-Jun phosphoylation in MDA-MB-468 cells.** NP-40 solubilized cell lysates were immunoblotted with anti-JNK[pS183] antibody (a) or anti-c-Jun[pS63] antibody (b) or anti-c-Jun antibody (c) as a loading control using MDA-MB-468 cells treated with no HA (lane 1) or with HA (50 μg/ml) for 15 min (lane 2) or pretreated with anti-CD44 antibody for 1 h followed by 15 min HA (50 μg/ml) addition (lane 3) or pretreated with JNK inhibitor (420116) (20 μM) for 1 h followed by 15 min HA (50 μg/ml) addition (lane 4) or treated with non-immune IgG without HA (lane 5) or treated with non-immune rat IgG plus HA (lane 6). [The ratio of phosphorylated c-JNK (a) or phosphorylated c-Jun (b) and c-Jun (the loading control) (c) was determined by densitometry, and the levels were normalized to untreated (without HA treatment) value or non-immune rat IgG (without HA treatment) (designated as 1.00); the values expressed represent an average of triplicate determination of 4 experiments with an SD of less than 5%].

Previous studies [1,25,30-33] indicated that HA/CD44-mediated oncogenic signaling plays an important role in the development of several solid tumors including breast cancer. Among the signaling aberrations present in breast cancer, JNK and c-Jun signaling activation appears to be one of the critical pathways for the development of breast cancer [34,35]. Gene regulation by JNK-mediated c-Jun signaling generally requires specific phosphorylation of these two molecules [36,37]. Specifically, JNK phosphorylates c-Jun at Ser-63[pS63] residues within the transcriptional activation domain of c-Jun [36]. In this study we focused on the question of whether HA can regulate JNK activation and c-Jun signaling in breast tumor cells. To this end we examined a HA-mediated phosphorylation of JNK and c-Jun. Using anti-phospho-JNK[pS183]- and anti-phospho-c-Jun[pS63]-mediated immunoblot or anti-c-Jun immunoblot, respectively, we observed that phosphorylation of both JNK and c-Jun occurs as early as 15 min following HA addition to MDA-MB-468 cells (Figure 1-lane 2). In contrast, only a relatively low level of phosphorylated JNK and c-Jun is present in cells pretreated with anti-CD44 antibody plus HA (Figure 1-lane 3) or without any HA treatment (Figure 1-lane 1). However, non-immune rat IgG does not appear to block HA-mediated JNK and c-Jun phosphorylation (lane 5 and 6). These results indicate that phosphorylation of JNK and c-Jun is HA-dependent and CD44-specific. Treatment of the JNK inhibitor (420116) (Figure 1-lane 4) also effectively reduces HA-mediated JNK and c-Jun phosphorylation (lane 5 and 6). These observations clearly indicate that activation of JNK and c-Jun is closely associated with HA-CD44 interaction in MDA-MB-468 cells.

### HA-CD44 binding promotes nuclear translocation of c-Jun in MDA-MB-468 cells

**Figure 2:**
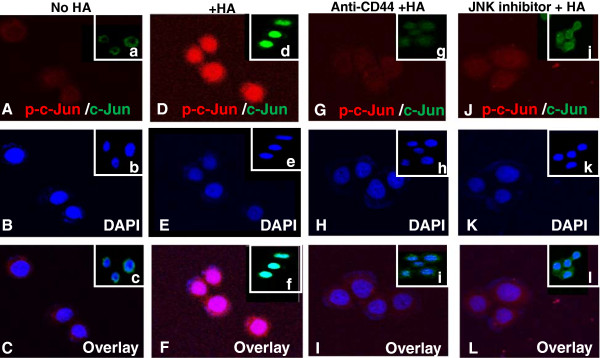
**Immunocytochemical analyses of HA/CD44-induced nuclear translocation of phospho-c-Jun/c-Jun in MDA-MB-468 cells.** MDA-MB-468 cells [untreated or pretreated with anti-CD44 antibody or JNK inhibitor (420116) (20 μM) for 1 h] were incubated with HA (50 μg/ml) (or without HA) for 30 minutes at 37°C and fixed by 2% paraformaldehyde. Subsequently, these cells were rendered permeable by ethanol treatment and immunostained with Texas Red-labeled anti-p-c-Jun (red color) **(A, D, G, J)** and DAPI (a nuclear marker) **(B, E, H, K)** or FITC-labeled anti-c-Jun (green color) (Insert-a, d, g, j) and DAPI (a nuclear marker) (Insert-b, e, h, k). [Overlay images represent simultaneous localization of Texas Red-labeled p-c-Jun (red color) and DAPI (a nuclear marker) **(C, F, I, L)** or FITC-labeled anti-c-Jun (green color) and DAPI (a nuclear marker) (c, f, i, l)].

HA/CD44-mediated nuclear translocation of transcription factors is reported in a number of previous studies [25,29,31,38]. In this study using immunofluorescence staining and confocal microscopy, we observed that both phosphorylated c-Jun[pS63] and c-Jun translocate from the cytosol to the nucleus after 30 min HA treatment (Figure 2A and 2B; Table 1). In contrast, most of the phosphorylated c-Jun[pS63] (Figure 2A) and total c-Jun (Figure 2B) is found in the cytosol, and only a low level of phosphorylated c-Jun/total c-Jun is present in the nucleus of MDA-MB-468 cells- either pretreated with anti-CD44 antibody plus HA (Figure 2A and 2B; Table 1) or without any HA treatment (Figure 2A and 2B). However, non-immune Rat IgG fails to reduce HA-mediated c-Jun and p-c-Jun nuclear accumulation (Table 1). These results indicate that nuclear translocation of c-Jun or p-c-Jun is HA-dependent and CD44-specific. We also noted that neither phosphorylated-c-Jun[pS63] nor total c-Jun undergoes nuclear translocation in cells treated with JNK Inhibitor, 420116 plus HA (Figure 2A and Figure 2B).

**Table 1:** Measurement of HA-induced p-c-Jun and c-Jun nuclear accumulation in MDA-MB-468 cells

**Treatments**	**p-c-Jun nuclear accumulation (% of control)**	**c-Jun nuclear accumulation (% of control)**
No HA (control)	100% ± 5	100% ± 3
+ HA	275% ± 13^a^	260% ± 10^b^
Non-immune IgG (No HA)	102% ± 4^a^	101% ± 5^b^
Non-immune IgG (+HA)	268% ± 9^a^	275% ± 11^b^
Anti-CD44 antibody + HA	92% ± 3^a^	87% ± 3^b^
Vehicle control + HA	262% ± 8^a^	279% ± 8^b^
JNK inhibitor + HA	84% ± 4^a^	75% ± 3^b^

p-c-Jun or c-Jun was detected in the nucleus which was stained with Texas Red-labeled anti-p-c-Jun antibody or FITC-labeled anti-c-Jun antibody (as shown in Figure 2). The labeling index for p-c-Jun or c-Jun was indeoendently determined by two observers and corresponded to the percentage of positive nuclear accumulatiohjn among 1,000 cells using Epi-fluorescence microscope (at 40X objectives).

All data represent mean ± SEM (with n = 4) of p-c-Jun and c-Jun nuclear accumulation activity detected in each sample.

^a, b^Statistically significant (*P* < 0.005; analysis of variance; n = 4) as compared with control samples (No HA treatment).

The reason for showing both phospho-c-Jun and total c-Jun in MDA-MB-468 cells following HA treatment is to determine whether phosphorylated c-Jun represents the majority or a minority species of total c-Jun. The fact that the JNK inhibitor prevents nuclear translocation of both phospho-c-Jun and Jun suggests that majority of c-Jun is phosphorylated by JNK. This explains the effect of JNK inhibitor on blocking both phosphorylated c-Jun and total c-Jun nuclear accumulation in cells treated with HA. These findings strongly suggest that the HA-CD44 interaction promotes c-Jun nuclear translocation in MDA-MB-468 cells in a JNK-dependent manner.

### Role of c-Jun in regulating miR-21 gene expression in HA/CD44

**Figure 3:**
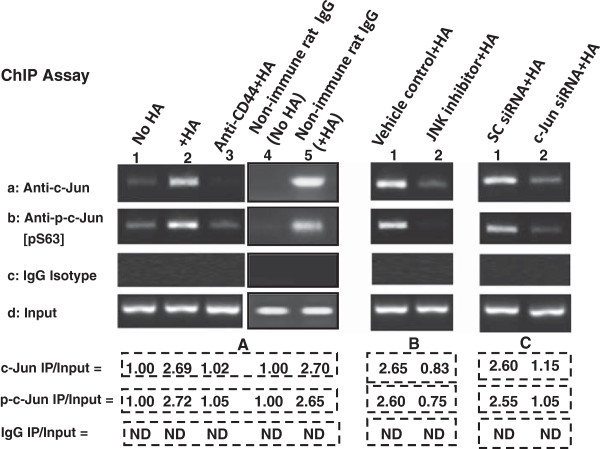
**Analyses of an *in vivo *binding of c-Jun (or phosphorylated c-Jun) to the miR-21 upstream promoter/enhancer region in MDA-MB-468 cells.** In order to analyze the interaction between c-Jun/phospho-c-Jun and the upstream promoter/enhancer region of miR-21 promoter in MDA-MB-468 cells, ChIP assay was performed in MDA-MB-468 cells following protocols described in Materials and methods using the AP1 binding site containing miR-21 promoter (upstream promoter/enhancer region)-specific primers by PCR. Identical volumes from the final precipitated materials were used for the PCR reactions [untreated cells (**A**: lane 1); cells treated with HA for 1 h (**A**: lane 2); cells pretreated with anti-CD44 antibody plus 1 h HA addition (**A**: lane 3); cells treated with non-immune rat IgG (without HA addition) (**A**: lane 4) or cells treated with non-immune rat IgG plus 1 h HA addition (**A**: lane 5); or cells treated with vehicle control plus 1h HA addition (**B**: lane 1) or treated with JNK inhibitor plus 1h HA addition (**B**: lane 2); or cells pretreated with scrambled siRNA plus 1h HA addition (**C**: lane 1); or cells pretreated with c-Jun siRNA plus 1h HA addition (**C**: lane 2)]. (a: anti-c-Jun-mediated immunoprecipitated material; b: anti-phospho-c-Jun[pS63]-mediated immunoprecipitated material; c: IgG isotype control-mediated precipitated material; d: total input materials). [The ratio of c-Jun IP materials (a) or phosphorylated c-Jun IP materials (b) or IgG IP materials (c) and total Input materials (the loading control) (d) was determined by densitometry, and the levels were normalized to untreated (without HA treatment) value or non-immune IgG (without HA treatment) (designated as 1.00); the values expressed represent an average of triplicate determination of 4 experiments with an SD of less than 5%]. ND represents Not Detectable.

A previous study indicated that miR-21 is regulated by an upstream/enhancer promoter containing AP1 binding sites [39]. To examine whether c-Jun directly interacts with the upstream/enhancer region of the miR-21 promoter, anti-c-Jun antibody or anti-phospho-c-Jun [pS63]-specific chromatin immunoprecipitation (ChIP) assays were performed on MDA-MB-468 cells. As shown in Figure 3, the PCR from anti-c-Jun or anti-phospho-c-Jun[pS63]-mediated precipitations from HA-treated MDA-MB-468 cells resulted in a specific amplification product using a primer pair specific for the miR-21 promoter/enhancer region containing the AP1 binding sites (Figure 3A-a, b-lane 2). In contrast, a reduced amount of the c-Jun or phospho-c-Jun binding of miR-21 upstream/enhancer promoter region was detected in cells pretreated with anti-CD44 antibody followed by HA addition (Figure 3A-a, b-lane 3), or without HA treatment (Figure 3A-a, b-lane 1). However, non-immune rat IgG does not appear to block HA-mediated c-Jun and p-c-Jun association with the miR-21 promoter (Figure 3A-a, b-lane 4 and 5). These findings suggest that the recruitment of c-Jun (also phospho-c-Jun) into the upstream/enhancer region of miR-21 promoter site is HA-specific and CD44-dependent.

To confirm the direct involvement of JNK-mediated c-Jun signaling in miR-21 gene upregulation, JNK activity was blocked by a JNK Inhibitor, 420116, (Figure 3B) and c-Jun was downregulated by c-Jun small interfering RNA (siRNA), (Figure 3C) followed by the miR-21 promoter-specific ChIP assay as described above. Our results indicate that (i) inhibition of c-JNK (but not vehicle control samples) (Figure 3B-a, b-lane 2 vs. lane 1) or (ii) transfection of MDA-MB-468 cells with c-Jun siRNAs (but not scrambled sequenced siRNA) (Figure 3C-a, b-lane 2 vs. lane 1) effectively blocked HA-mediated c-Jun/phospho-c-Jun binding to the miR-21 upstream/enhancer promoter region with AP1 binding sites in MDA-MB-468 cells. Identical amplification products were detected in the positive controls from total input chromatin (Figure 3A, B, C-d-all lines). Moreover, no amplification was seen in samples that were processed by IgG isotype control-mediated precipitation (Figure 3A, B, C-c-all lanes). Therefore, we concluded that downregulation of JNK activity or c-Jun/phospho-c-Jun expression by either JNK inhibitor (420116) or c-Jun siRNA is specific.

### HA-CD44-activated JNK/c-Jun signaling stimulates miRNA-21 production in MDA-MB-468 Cells

**Figure 4:**
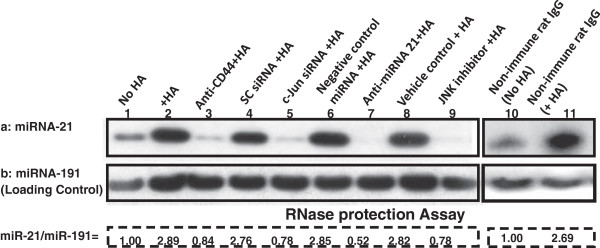
**Detection of HA/CD44-induced miR-21 production in MDA-MB-468 cells.** Detection of miR-21 in MDA-MB-468 cells using RNase protection assay as described in Materials and methods. a: Autoradiogram of miR-21 detected in MDA-MB-468 cells incubated without HA (a: lane 1) or with 2 h HA treatment (a: lane 2) or pretreated with anti-CD44 antibody for 1 h followed by HA addition for 2 h (a: lane 3) or incubated with scrambled siRNA plus 2 h HA treatment (a: lane 4) or c-Jun siRNA plus 2 h HA treatment (a: lane 5) or incubated with negative miRNA control plus 2 h HA treatment (a: lane 6) or incubated with anti-miRNA-21 plus 2 h HA treatment (a: lane 7) or 2 hours HA treatment (a: lane 8) or pretreated cells with JNK Inhibitor (420116) plus 2 h HA treatment (a: lane 9) or treated with non-immune IgG (without HA addition) (lane 10) or treated with non-immune IgG (with HA addition for 2 h). [Autoradiogram of miR-191 (b) in each gel lane was used as a loading control]. [The ratio of miRNA-21 (a) and miRNA-191 (the loading control) (b) was determined by densitometry, and the levels were normalized to untreated (without HA treatment) value or non-immune IgG (without HA treatment) (designated as 1.00); the values expressed represent an average of triplicate determination of 4 experiments with an SD of less than 5%].

The expression of mature miR-21 is involved in breast cancer progression [31,40]. To determine whether miR-21 levels are increased following the binding of HA to CD44, we first prepared small RNAs followed by an RNase protection assay using the miRNA Detection Kit (Ambion). Our results indicated that the level of miR-21 is definitely increased in MDA-MB-468 cells treated with HA (Figure 4a-lane 2) compared with those cells not treated with HA (Figure 4a-lane 1) or with anti-CD44 antibody treatment plus HA addition (Figure 4a-lane 3). However, non-immune rat IgG does not inhibit HA-mediated miR-21 production (lane 10 and 11). These results indicate that miR-21 expression is HA-dependent and CD44-specific in MDA-MB-468 cells. Transfection of these cells with c-Jun siRNA (Figure 4a-lane 5) caused significantly less HA-induced miR-21 expression compared to those cells treated with scrambled siRNA (Figure 4a-lane 4). These findings support the notion that c-Jun is required for miR-21 production in HA-activated MDA-MB-468 cells. Moreover, we found that the expression of miR-21 can be induced in cells treated with a miRNA-negative control upon addition of HA (Figure 4a-lane 6). In contrast, the treatment of MDA-MB-231 cells with an anti-miR-21 inhibitor plus HA resulted in a decrease in miR-21 expression (Figure 4a-lane 7). Treatment of MDA-MB-468 cells with a JNK inhibitor (420116) also significantly attenuated HA-CD44-induced miR-21 production as compared to vehicle-treated cells with HA addition (Figure 4a-lane 8 and lane 9). We believe that these changes in miR-21 expression under various treatment conditions are not due to the variations of RNA extracted from each sample since there were very similar levels of miR-191 (as a loading control) in all samples (Figure 4b-lane 1-9). Together, these findings strongly indicate that HA/CD44-activated JNK/c-Jun signaling plays an important role in the production of miR-21 in breast tumor cells.

### The impact of HA/CD44-mediated miR-21 (induced by c-Jun signaling) on Bcl2/IAP expression, anti-apoptosis and chemoresistance in MDA-MB-468 cells

**Figure 5:**
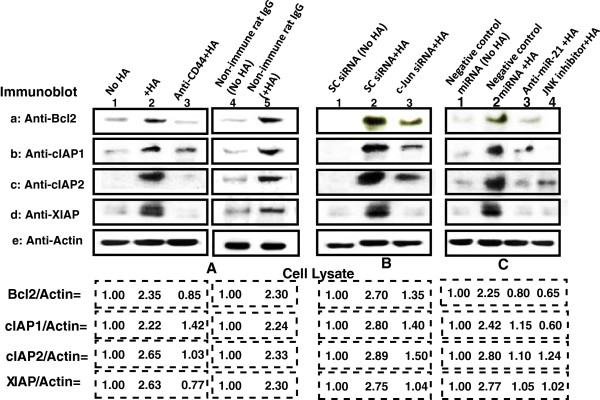
**Analyses of HA/CD44-mediated Bcl-2 and IAP expression in MDA-MB-468 cells. (A)** Cell lysates from cells [treated with no HA (lane 1) or with HA (lane 2) or pretreated with anti-CD44 antibody plus HA (lane 3) or treated with non-immune IgG (without HA) (lane 4) or with HA (lane 5)] were immunoblotted with anti-BCL-2 antibody (a) or anti-cIAP-1 antibody (b) or anti-cIAP-2 antibody (c) or anti-XIAP antibody (d) or anti-actin antibody (e), respectively. [The ratio of Bcl2 (a) or cIAP1 (b) or cIAP2 (c) or XIAP (d) and actin (c) was determined by densitometry, and normalized to untreated (without HA treatment) value or non-immune IgG (no HA) (designated as 1.00)]. **(B)** Cell lysates from cells [transfected with scrambled siRNA with no HA (lane 1) or with HA (lane 2) or transfected with c-Jun siRNA plus HA (lane 3)] were immunoblotted with anti-BCL-2 antibody (a) or anti-cIAP-1 antibody (b) or anti-cIAP-2 antibody (c) or anti-XIAP antibody (d) or anti-actin antibody (e), respectively. [The ratio of Bcl2 (a) or cIAP1 (b) or cIAP2 (c) or XIAP (d) and actin (e) was determined by densitometry, and normalized to the value of scrambled siRNA-treated samples (no HA) (designated as 1.00)]. **(C)** Cell lysates from cells [transfected with negative miRNA control plus no HA (lane 1) or with HA (lane 2) or transfected with anti-miRNA-21 plus HA (lane 3) or treated with JNK inhibitor plus HA (lane 4)] were immunoblotted with anti-cIAP-1 antibody (a) or anti-cIAP-2 antibody (b) or anti-XIAP antibody (c) or anti-actin antibody (e), respectively. [The ratio of Bcl2 (a) or cIAP1 (b) or cIAP2 (c) or XIAP (d) and actin (e) was determined by densitometry, and normalized to the value of negative control miRNA-treated samples (no HA) (designated as 1.00)]. The values expressed represent an average of triplicate determination of 4 experiments with an SD of less than 5%.

Bcl-2 is identified as one of the target proteins induced by miR-21 [24]. Inhibitors of the apoptosis family of proteins (IAPs) [cIAP-1, cIAP-2 and X-linked IAPs (XIAP)] are frequently overexpressed by cancer cells. Importantly, upregulation of IAPs is linked to chemoresistance due to binding to caspases and suppressing apoptosis [41]. Here, we demonstrated that the expression of both Bcl2 and IAPs (cIAP1/cIAP2/XIAP) is greatly enhanced in cells treated with HA (Figure 5A-a, b, c, d-lane 2). In contrast, low basal levels of Bcl2 expression and IAPs (cIAP1/cIAP2/XIAP) exist in untreated cells (Figure 5A-a, b, c, d-lane 1) or cells pretreated with anti-CD44 antibody followed by HA addition (Figure 5A-a, b, c, d-lane 3). The fact that non-immune rat IgG fails to block HA-mediated Bcl2 and IAPs (cIAP1/cIAP2/XIAP) expression (Figure 5A-a, b, c, d-lane 4 and 5) suggests that up-regulation of Bcl2 and IAPs (cIAP1/cIAP2/XIAP) is HA-dependent and CD44-specific. Further analyses indicated that the expression of both Bcl2 and IAPs is significantly downregulated in MDA-MB-468 cells treated with c-Jun siRNA (Figure 5B-a, b, c, d-lane 3) or JNK inhibitor (Figure 5C-a, b, c, d-lane 4). However, both Bcl2 and IAPs are up-regulated in MDA-MB-468 cells treated with scrambled sequence siRNA in the presence of HA (Figure 5B-a, b, c, d-lane 2) as compared to no HA addition (Figure 5B-a, b, c, d-lane 2). These results suggest that HA/CD44-mediated JNK/c-Jun signaling is closely linked to the expression of Bcl2 and/or IAPs in MDA-MB-468 cells. Moreover, we noted that downregulation of miR-21 by treating MDA-MB-468 cells with an anti-miR-21 inhibitor (but not a negative-control miRNA) promotes downregulation of Bcl2 and IAPs (cIAP1/cIAP2/XIAP) (Figure 5C-a, b, c, b-d, lane 3 vs. lane 1 and lane 2) in the presence of HA. These results indicate that the signaling network consisting of JNK/c-Jun signaling and miR-21 is functionally coupled with the up-regulation of anti-apoptosis and survival protein (e.g., Bcl2, cIAP1/cIAP2/XIAP) production. These specific effects likely facilitate the oncogenic progression of HA/CD44-activated MDA-MB-468 cells.

Finally, further analyses showed that the addition of HA enhances cell growth/survival and reduces apoptosis in untreated control cells or anti-CD44 antibody treated cells (but not non-immune rat IgG treated cells) (i.e., without chemotherapeutic drugs) and decreases the ability of Doxorubicin to induce tumor apoptosis and cell death (Table 2A). These observations indicated that HA causes both a decrease in apoptosis and an increase in breast tumor cell growth and survival (Table 2A & B) leading to the enhancement of chemoresistance (Table 2A). Moreover, downregulation of c-Jun or miR-21 [by treating MDA-MB-468 cells with c-Jun siRNA, JNK inhibitor or transfected MDA-MB-468 cells with an anti-miR-21 inhibitor (but not scrambled sequence siRNA or with miRNA-negative control)] effectively attenuates HA-mediated tumor cell growth/anti-apoptosis/survival and enhances chemotherapy sensitivity in MDA-MB-231 cells (Table 2A & B). Taken together, these findings strongly suggest that the HA/CD44-mediated JNK/c-Jun signaling pathways and miR-21 function represent new treatment targets to force tumor cells to undergo apoptosis/death and to overcome chemotherapy resistance in breast cancer cells.

**Table 2:** Measurement of doxorubicin-induced MDA-MB-468 cell apoptosis and growth inhibition

***Treatments***	Apoptotic cells (Annexin V-positive cell/total cells × 100%)*		**Doxorubicin-induced tumor cell growth inhibition IC**_**50**_(μM)**
	**No doxorubicin**	**+ doxorubicin**	
(A) Effects of various signaling perturbation agents on doxorubicin--induced apoptosis and cell growth inhibition in MDA-MB-468 cells.			
(a) Effects of Doxorubicin-induced apoptosis and cell growth inhibition in MDA-MB-468 cells following HA treatment:			
No HA (Untreated cells)	4.1 ± 0.5	30.2 ± 3.1^a^	73.5 ± 5.1
+ HA	2.4 ± 0.3^a^	12.6 ± 2.3^a^	188.0 ± 10.5^b^
Non-immune rat IgG-treated cells (No HA)	3.8 ± 0.5^a^	29.2 ± 2.2^a^	70.3 ± 4.1^b^
Non-immune rat IgG-treated cells (+ HA)	2.2 ± 0.2^a^	10.5 ± 2.0^a^	176.0 ± 10.3^b^
Anti-CD44-treated cells (+ HA)	3.8 ± 0.2^a^	29.2 ± 2.2^a^	28.4 ± 3.6^b^
(b) Effects of Doxorubicin-induced apoptosis and cell growth inhibition in MDA-MB-468 cells treated with c-Jun siRNA plus HA:			
Scrambled siRNA-treated cells (No HA)	5.2 ± 0.6	32.4 ± 3.6^c^	85.0 ± 2.4
Scrambled siRNA-treated cells (+ HA)	4.5 ± 0.5^c^	11.9 ± 3.1^c^	190.0 ± 6.00^d^
c-Jun siRNA-treated cells (No HA)	5.2 ± 0.6^c^	33.6 ± 2.8^c^	40.5 ± 0.30^d^
c-Jun siRNA-treated cells (+ HA)	4.5 ± 0.5^c^	31.9 ± 3.2^c^	39.2 ± 0.80^d^
(c) Effects of Doxorubicin-induced apoptosis and cell growth inhibition in MDA-MB-468 cells treated with anti-miR-21 inhibitor plus HA:			
miRNA negative control-treated cells (No HA)	3.7 ± 0.4	31.6 ± 2.9^e^	62.8 ± 0.5
miRNA negative control-treated cells (+ HA)	2.5 ± 0.2^e^	12.3 ± 0.4^e^	196.0 ± 4.2^f^
Anti-miR-21 inhibitor-treated cells (No HA)	3.2 ± 0.6^e^	38 ± 2.5^e^	65.0 ± 3.0^f^
Anti-miR-21 inhibitor-treated cells (+ HA)	3.1 ± 0.4^e^	34 ± 2.1^e^	70.0 ± 0.2^f^
(B) Effects of JNK inhibitor (420116) on Doxorubicin-induced apoptosis and cell growth inhibition in MDA-MB-468 cells.			
Vehicle control-treated cells (No HA)	4.3 ± 0.2	32 ± 2.4^g^	64.00 ± 6.2
Vehicle control-treated cells (+ HA)	2.6 ± 0.4^g^	14 ± 0.5^g^	180.0 ± 10.0^h^
JNK inhibitor (420116)-treated cells (No HA)	12.0 ± 0.5^g^	35 ± 2.6^g^	26.0 ± 3.5^h^
JNK inhibitor (420116)-treated cells (+ HA)	3.2 ± 0.3^g^	38 ± 3.3^g^	25.0 ± 0.2^h^

*Cells were designated apoptotic when displaying Annexin V-positive staining. In each sample, at least 500 cells from five different fields were counted, with the percentage of Doxorubicin or JNK inhibitor (420116) (2 h treatment)-induced apoptotic cells calculated as Annexin V-positive cells/total number of cells. The values are presented as the means ± standard deviation. All assays consisted of at least six replicates and were performed on at least 4 different experiments.

**Tumor cell growth inhibition (IC_50_) is designated as “the μM concentration of chemotherapeutic drug (e.g., Doxorubicin-24 h treatment) that causes 50% inhibition of tumor cell growth” using MTT-based growth assay as described in the Materials and methods. IC_50_ values are presented as the means ± standard deviation. All assays consisted of at least six replicates and were performed on at least 4 different experiments.

^a, b^Statistically significant (*P* < 0.005; analysis of variance; n = 4) as compared with control samples (No HA treatment).

^c, d^Statistically significant (*P* < 0.005; analysis of variance; n = 4) as compared with control samples (scrambled siRNA-treated cells without HA addition).

^e, f^Statistically significant (*P* < 0.005; analysis of variance; n = 4) as compared with control samples (miRNA negative control-treated cells without HA addition).

^g, h^Statistically significant (*P* < 0.001; analysis of variance; n = 4) as compared with control samples (vehicle control-treated cells without HA addition).

## Discussion

Hyaluronan (HA) is an important structural component of the extracellular matrix (ECM). In cancer patients, the level of HA is usually higher in malignant tumors than in corresponding benign or normal tissues, and in some tumor types the level of HA is predictive of malignancy [2-7,42]. In particular, HA level is elevated in the serum of breast cancer patients [4,42]. The aberrant HA production by HA synthases [43-45] and HMW-HA degradation into LMW-HA by hyaluronidases [46] are thought to be closely associated with breast tumor cell progression [4].

HA binds specifically to CD44, a family of multifunctional transmembrane glycoproteins expressed in numerous cells and tissues, including breast tumor cells and various carcinoma tissues [2-4]. The crystal structure of the HA-CD44 complex was reported previously and a single HA binding site was identified [47]. CD44 is generally expressed in a variety of isoforms that are products of a single gene generated by alternative splicing of variant exons inserted into an extracellular membrane-proximal site [48,49]. CD44 is also expressed in tumor stem cells that have the unique ability to initiate tumor cell-specific properties [38,50]. In fact, CD44 is considered to be one of the important surface markers on cancer stem cells [38,50]. HA binding to CD44 is involved in the stimulation of both receptor kinases (e.g., ErbB2, EGFR and TGFβ receptors) and non-receptor kinases (e.g., c-Src and ROK) [3] required for a variety of tumor cell-specific functions leading to tumor progression.

Abnormal JNK/c-Jun signaling also appears to play a critical role in oncogenesis [34,35]. JNK-activated c-Jun is a signal-transducing transcription factor of the AP-1 family that is implicated in cell cycle progression, differentiation and cell transformation [51]. It has a direct role in regulating the transcription of p53 and cyclinD1 [52,53]. It has also been shown that c-Jun accelerates leukemogenesis and regulates the activation of genes required for cell cycle progression in tumor cells [51]. The AP-1 factor c-Jun is thought to act as a “bodyguard”, preventing methylation of a distinct set of genes after oncogenic transformation [51]. Recently, c-Jun is found to trigger miR-21 transcription through AP-1 binding sites present in the miR-21 promotor region [39]. In this study we observed that HA-CD44 binding results in c-Jun (also causes phosphorylation of c-Jun) nuclear localization in MDA-MB-468 cells (Figure 2). Thus, identifying specific genes that are transcriptionally controlled by the JNK/c-Jun signaling during HA-CD44 interaction in the nucleus may be essential for understanding the disease mechanism occurring during breast cancer progression.

Overexpression of miR-21 is detected in various breast cancer cell lines and patient specimens [25,26]. Accumulating evidence indicates that miR-21 is closely associated with both cancer development and chemotherapy resistance [25]. The stem cell marker, Nanog, has been found to be involved in the regulation of pri-miRNA expression during cancer development [25]. Our previous work indicated that HA/CD44-activated PKCϵ promotes Nanog interaction with p68 and DROSHA leading to biosynthetic processing and production of miR-21 in breast tumor cells [25]. These findings suggest that HA/CD44-mediated Nanog signaling is closely linked to miR-21 production and function during oncogenesis.

In this study, we provided new evidence that miR-21 expression is controlled by an upstream promoter/enhancer containing AP-1 binding sites in MDA-MB-468 cells while chromatin immunoprecipitation (ChIP) assays demonstrate that stimulation of miR-21 production by HA is JNK and c-Jun-dependent in breast tumor cells (Figure 4). Most importantly, downregulation of JNK/c-Jun signaling (by treating cells with JNK inhibitor or c-Jun siRNA) or miR-21 (by treating cells with anti-miR-21 inhibitor) reduces the expression of the target protein, Bcl2, and anti-apoptotic proteins [e.g., IAPs (cIAP1/cIAP2/XIAP)] (Figure 5) in breast tumor cells. Determining the cellular and molecular mechanisms involved in the regulation of these causal links between JNK/c-Jun signaling and miR-21 function, including Bcl2 and IAP upregulation, awaits further investigation.

Chemotherapy resistance is one of the primary causes of morbidity in patients diagnosed with solid tumors including breast cancer [54-56]. Chemotherapeutic agents, such as doxorubicin, are commonly used to inhibit DNA synthesis in the treatment of breast cancer patients [57]. In particular, the ability of doxorubicin to bind to DNA and/or produce free radicals is thought to be the mechanism for the induction of cytotoxic effects on tumor cells [57]. However, this drug often displays limited cytotoxic killing and anti-tumor effects due to chemoresistance which occurs in *de novo* tumor cells [57].

It is now certain that a number of oncogenic signaling pathways are closely involved with chemotherapeutic drug resistant phenotypes [25,29-31,38,58,59]. In particular, matrix HA interaction with CD44 in cancer cells have been strongly implicated in the development of chemoresistance [25,29-31,38,58,59]. Specifically, HA is capable of stimulating MDR1 (P-gp) expression and drug resistance in breast tumor cells [30,58,59]. CD44 also interacts with MDR1 (P-gp) to promote cell migration and invasion of breast tumor cells [30,58,59]. Previously we reported that activation of HA-CD44-mediated oncogenic signaling events [e.g., mir-302/miR-21, intracellular Ca^2+^ mobilization, epidermal growth factor receptor (EGFR)-mediated ERK signaling, topoisomerase activation, and ankyrin-associated cytoskeleton function] leads to multidrug resistance in a variety of tumor cells [3,10,33]. These observations strongly suggest a functional link between HA-mediated CD44 signaling and drug resistance.

In this study we demonstrated that HA/CD44-activated JNK/c-Jun signaling and miR-21 increases survival protein, Bcl2, resulting in oncogenesis by enhancing the expression of inhibitors of anti-apoptosis proteins (IAPs) (Figure 5). Furthermore, downregulation of HA/CD44-activated JNK/c-Jun signaling (by JNK I nhibitor/c-Jun siRNA) and miR-21 production (by anti-miR-21 inhibitor) not only reduces Bcl2 upregulation (Figure 5), but also inhibits the expression of survival proteins (e.g., c-IAP1, c-IAP2 and XIAP) (Figure 5). Consequently, these signaling perturbation events contribute to apoptosis and chemosensitivity (Table 1). Furthermore, this newly-discovered HA/CD44-activated JNK/c-Jun signaling pathway and miR-21 production/function should provide important new drug targets to cause tumor cell apoptosis and overcome chemotherapy resistance in breast tumor cells.

**Figure 6:**
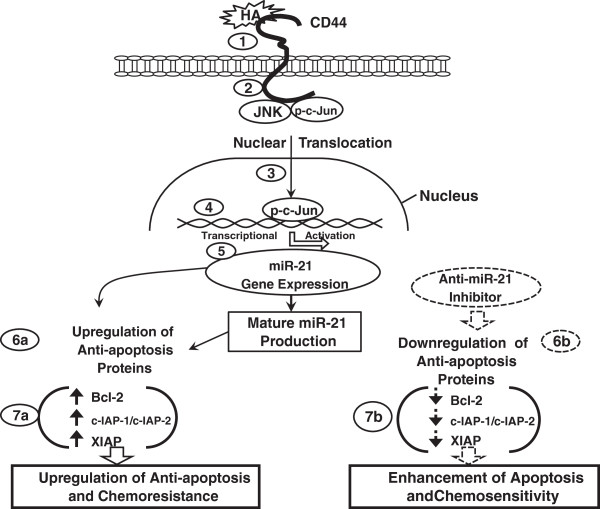
**A proposed model for HA/CD44-mediated JNK/c-Jun signaling in the regulation of miRNA-21 production, oncogenesis and chemoresistence in MDA-MB-468 cells.** We propose that HA-CD44 binding (step 1) promotes JNK and c-Jun (also serine phosphorylated JNK/c-Jun) (step 2). Subsequently, c-Jun (also p-c-Jun) translocates from the cytosol to the nucleus and interacts with an upstream/enhancer region (containing AP1 binding sites) of the miR-21 promoter (step 3), resulting in miR-21 gene expression (step 4) and mature miR-21 production (step 5). The resultant miR-21 then functions to upregulate the survival protein, Bcl2 (step 6a) and promotes MDA-MB-468 cell activation leading to IAP (c-IAP-1, c-IAP-2 and XIAP) expression (step 7a), MDA-MB-468 cell anti-apoptosis/survival and chemoresistance. In direct contrast, treatment of MDA-MB-468 cells with an anti-miR-21 inhibitor reduces Bcl2 upregulation (step 6b). Subsequently, these changes result in the inhibition of IAP (c-IAP-1, c-IAP-2 and XIAP) expression (step 7b), stimulation of apoptosis and enhancement of chemosensitivity in MDA-MB-468 cells. Taken together, these findings suggest that targeting HA/CD44-mediated JNK/c-Jun signaling pathways and miR-21 function may provide a new drug target to sensitize tumor cell apoptosis/death and overcome chemotherapy resistance in MDA-MB-468 breast tumor cells.

In summary (as shown in Figure 6), we propose that HA-CD44 binding (Step 1) promotes JNK and c-Jun (also serine phosphorylated JNK/c-Jun) (step 2). Subsequently, c-Jun (also p-c-Jun) translocates from the cytosol to the nucleus and interacts with an upstream/enhancer region (containing AP1 binding sites) of the miR-21 promoter (step 3), resulting in miR-21 gene expression (step 4) and mature miR-21 production (step 5). The resultant miR-21 then functions to upregulate the survival protein, Bcl2 (step 6a) and promotes MDA-MB-468 cell activation leading to IAP (c-IAP-1, c-IAP-2 and XIAP) expression (step 7a), MDA-MB-468 cell anti-apoptosis/survival and chemoresistance. In direct contrast, treatment of MDA-MB-468 cells with an anti-miR-21 inhibitor reduces Bcl2 upregulation (step 6b). Subsequently, these changes result in the inhibition of IAP (c-IAP-1, c-IAP-2 and XIAP) expression (step 7b), stimulation of apoptosis and enhancement of chemosensitivity in MDA-MB-468 cells. Taken together, these findings strongly suggest that targeting HA/CD44-mediated JNK/c-Jun signaling pathways and miR-21 function may provide new drug targets to sensitize tumor cell apoptosis/death and overcome chemotherapy resistance in MDA-MB-468 breast tumor cells.

## Materials and methods

### Cell culture

The cell line, MDA-MB-468 cells from ATCC, was isolated in 1977 by R. Cailleau, et al., from a pleural effusion of a 51-year-old Black female patient with metastatic adenocarcinoma of the breast. This cell line was cultured in ATCC-formulated Leibovitz’s L-15 Medium, Catalog No. 30-2008, with 10% fetal bovine serum.

### Antibodies and reagents

Monoclonal rat anti-CD44 antibody (Clone: 020; Isotype: IgG2b; obtained from CMB-TECH Inc., San Francisco, CA, USA) recognizes a determinant of the HA-binding region common to CD44 and its principal variant isoforms. This rat anti-CD44 was routinely used for HA-related blocking experiments. Immunoreagents such as rabbit anti-C-JUN antibody, mouse anti-Bcl-2 antibody and goat anti-actin antibody were purchased from Santa Cruz Biotechnology, Inc. (Santa Cruz, CA, USA). Mouse anti-c-IAP-1 antibody, mouse anti-c-IAP-2 and mouse anti-XIAP antibody were from BD (Franklin Lakes, NJ, USA). Rabbit anti-phospho-c-Jun [pS63] antibody, rabbit anti-c-Jun antibody, rabbit anti-JNK[pS63] antibody and rabbit anti-JNK antibody were from Cell Signaling Technology (Beverly, MA, USA). JNK Inhibitor I, 420116 was purchased from EMD Millipore (Billerica, MA, USA). Doxorubicin hydrochloride was from Sigma Chemicals (St. Louis, MO). Healon HA polymers (~500,000-dalton polymers), purchased from Pharmacia & Upjohn Co. (Kalamazoo, MI), were prepared as described previously [29,31,38].

### Anti-miR-21 inhibitor preparation and transfection

MDA-MB-468 cells were transfected with anti-miR-21 inhibitor (Ambion, catalog number, 17000) (30 nmol/l) and its corresponding miRNA negative control (Ambion, catalog number, 17010) (30 nmol/l) using Lipofectamine 2000 reagent (Invitrogen) for 24 hours. Cells were then treated with HA or no HA in various experiments as described below.

### Immunoblotting techniques

The NP-40 solubilized cell lysate materials from MDA-MB-468 cells [untreated or pretreated with anti-CD44 antibody or JNK Inhibitor I, 420116 (20 μM) or vehicle control] plus 50 μg/ml HA (or no HA) for various time intervals (e.g. 0, 5 min, 15 min or 60 min or 24 h) at 37°C] were immunoblotted with rabbit anti-c-Jun antibody (2 μg/ml) or rabbit anti-phospho-c-Jun [pS63] antibody (2 μg/ml) or rabbit anti-c-JNK[pS183] antibody (2 μg/ml), respectively. In some cases, cell lysate of MDA-MB-468 cells (transfected with c-Jun siRNA or siRNA with scrambled sequences; or anti-miR-21 inhibitor or miRNA-negative control; or without any treatment) followed by HA (50 μg/ml) addition (or no HA or anti-CD44 antibody pretreatment plus HA) at 37°C were also immunoblotted using various immuno-reagents (e.g., mouse anti-Bcl-2 (2 μg/ml) or mouse anti-c-IAP-1 or mouse anti-c-IAP-2 antibody and anti-XIAP (2 μg/ml) or goat anti-actin (2 μg/ml) (as a loading control), respectively.

### Chromatin immunoprecipitation assay

To examine whether c-Jun or phospho-c-Jun directly interacts with the upstream promoter/enhancer region (containing AP-1 binding site) of miR-21, chromatin immunoprecipitation (ChIP) assays was performed in MDA-MB-468 cells [pretreated with anti-CD44 antibody or JNK Inhibitor I, 420116 (20 μM)/vehicle control or transfected with c-Jun siRNA or siRNA with scrambled sequences] treated with HA (50 μg/ml) or without HA using a kit (EZ ChIP) from Millipore Corp according to the manufacturer’s instructions. Crosslinked chromatin lysates were sonicated and diluted with ChIP sonication buffer plus protease inhibitors, divided and incubated with normal rabbit IgG or rabbit anti-c-Jun antibody or rabbit phospho-c-Jun[pS63] antibody at 4°C overnight, then precipitated with protein G agarose. Crosslinking was reversed by overnight at 65°C incubation; DNA fragments were then extracted with PCR purification kit, analyzed by PCR and quantitated by PCR using primer pairs specific for the miR-21 upstream promoter/enhancer region containing the c-Jun binding sites: forward primer: 5′-TGGATAAGGATGACGCACAG-3′ and reverse primer: 5′-TGGTTTGAACCAATTAATAAGGAAA-3′ on an agarose gel as described previously [25,29,38,60].

### RNase protection assay analysis of mature miRNAs

Expression of miRNAs was qualitatively analyzed by RNase protection assay. For RNase protection assay, enriched small RNA isolated from MDA-MB-468 cells [transfected with scrambled sequence siRNA with (or without) anti-CD44 antibody or JNK Inhibitor I, 420116 (20 μM)/vehicle control or transfected with c-Jun siRNA or siRNA or anti-miR-21 inhibitor or miRNA-negative control in the presence or absence of HA for various time intervals (e.g., 0, 5 min, 10 min, 15 min, 30 min or 2 h) at 37°C] was enriched and purified using the *mirVana* miRNA Isolation kit (Ambion). RNA concentrations were verified by measuring absorbance (A_260_) on the NanoDrop Spectrophotometer ND-1000 (NanoDrop). The *mirVana* miRNA probe construction kit (Ambion) was used to synthesize the ^32^P-labeled miR-21 antisense probe and miR-191 probe loading control as described previously [25,29,38].

### Immunofluorescence staining

MDA-MB-468 cells (untreated or pretreated with anti-CD44 antibody) were incubated with HA (50 μg/ml) at 37°C for 30 minutes or with no HA. These cells were then fixed with 2% paraformaldehyde. Subsequently, these cells were rendered permeable by ethanol treatment followed by incubating with Texas Red-conjugated anti-phospho-c-Jun[pS63] antibody or fluorescein (FITC)-conjugated anti-c-Jun antibody followed by DAPI staining (a marker for nucleus). These fluorescence-labeled samples were then examined with a confocal laser scanning microscope.

### Tumor cell growth and apoptosis assays

MDA-MB-468 cells were either untreated or pretreated with anti-CD44 antibody or treated with JNK inhibitor, 420116 (20 μM) or transfected with c-Jun siRNA or siRNA with scrambled sequences or anti-miR-21 or miRNA-negative control] in the presence or absence of 50 μg/ml HA, as above. These cells were then plated in 96-well culture plates in 0.2 ml of Dulbecco’s modified Eagle’s medium/F12 medium supplement (GIBCO, Grand Island, NY) containing no serum for 24 h at 37°C in 5% CO_2_/95% air. These cells (5 × 10^3^ cells/well) were then incubated with various concentrations of Doxorubicin (4 × 10^−9^ M-1.75 × 10^−5^ M) with no HA or with HA (50 μg/ml). After 24h incubation at 37°C, MTT-based growth assays were analyzed as described previously [25,30]. The percentage of absorbance relative to untreated controls (i.e., cells treated with neither HA nor chemotherapeutic drugs) was plotted as a linear function of drug concentration. The 50% inhibitory concentration (IC_50_) was identified as a concentration of drug required to achieve a 50% growth inhibition relative to untreated controls. For apoptosis assay, FITC-conjugated Annexin V (for measuring apoptotic cells) using Apoptosis Detection Kit (Calbiochem, San Diego, CA) was used according to the manufacturer’s protocol.

## Competing interests

All authors declare that they have no competing interest.

## Authors’ contributions

LC performed the biochemical, molecular and immunofluorescence experiments and drafted the manuscript. LYWB conceived the idea, supervised the data collections/analysis and finalized the presentation of the manuscript. Both authors read and approved the final manuscript.
